# Clinical and molecular characteristics of triple-negative breast cancer patients in Northern Israel: single center experience

**DOI:** 10.1186/s40064-015-0900-3

**Published:** 2015-03-15

**Authors:** Karama Asleh-Aburaya, Georgeta Fried

**Affiliations:** Division of Oncology, Rambam Health Care Campus, HaAliya Street 8, Haifa, 35254 Israel; The Ruth and Bruce Rappaport Faculty of Medicine, Technion-Israel Institute of Technology, Efron St. 1, Haifa, 31096 Israel

**Keywords:** Triple Negative Breast Cancer, BRCA mutation, Overall Survival, Distant metastasis

## Abstract

**Introduction:**

Triple-negative breast cancer (TNBC) lacks estrogen and progesterone receptors and does not overexpress HER2. It displays a distinct clinical behavior. This study aims to assess the clinical, molecular and prognostic characteristics of TNBC patients.

**Patients/Methods:**

TNBC patients, referred to a tertiary medical center, 1/1/2000 – 31/12/2005, were included. Clinical, molecular and prognostic characteristics were retrospectively collected from patients’ records.

**Results:**

Overall, 122 consecutive TNBC patients were included with a median age of 54 years. Among the TNBC patients, 101 (82.8%) were Jews and 21 (17.2%) were Arabs.

Family history for breast cancer was reported in 30 patients (24.6%). Genetic counseling was conducted in 30 patients (24.6%); 22/30 (73.3%) had BRCA1/2 mutations.

Median tumor size was 2 cm and positive lymph nodes were detected in pathological examination in 40 patients (34%). At the time of data analysis, 21/118 patients (17.8%), who initially presented with early disease, had developed metastasis. Local recurrence was detected in four patients (3.4%). The overall survival (OS) was significantly longer for patients younger than 60 years compared to those ≥ 60 years, (Hazard ratio (HR) =2.1, p=0.046). Nulliparous patients had significantly higher OS than patients with a reproductive history of ≥ 4 children. (HR=0.31, p= 0.041). Mortality rate was higher for Arabs versus Jews but did not reach significance, (HR=1.33; P=0.64).

**Conclusions:**

TNBC represents an exclusive clinical behavior. Older age and parity were found to be poor prognostic factors. Further larger studies are needed to reaffirm our findings and explore the genetics among non-BRCA1/2 TNBC patients.

## Introduction

Breast cancer is the most commonly occurring female malignancy and the second most common cause of death from cancer (DeSantis et al. 2011). It is well-established that breast cancer is a heterogeneous disease that encompasses various histopathological subtypes according to the Perou-Sorlie classification. This classification identifies different immunohistochemical biomarkers: estrogen receptor (ER), progesterone receptor (PR), human epidermal growth factor receptor (HER) 2, and the Ki-67 proliferation index (Sørlie et al. 2001). It identifies subtypes with a distinct clinical course and prognosis (Hammond et al. 2010).Triple-negative breast cancer (TNBC) is negative for ER, PR and HER2 (Ribelles et al. 2013).

TNBC constitutes approximately 10–20% of breast cancer patients and represents an aggressive subtype with a poor overall prognosis (Carey et al. 2010; Foulkes et al. 2010). Moreover, TNBC patients have a higher rate of early recurrence and distant metastasis to brain and lungs compared to other breast cancer subtypes (Carey et al. 2010). A high percentage of women with TNBC (34%) relapse within the first three years of follow-up (Dent et al. 2007).

In addition to histopathological subtypes, the clinical course might be affected by the molecular genetic profile, including the germ-line mutation of the genes BRCA1 and BRCA2 (Badve et al. 2011). The interplay between BRCA mutations and TNBC is complex. Most breast tumors displaying BRCA1 mutations are TNBC (Atchley et al. 2008). Furthermore, BRCA1-deficient tumors share biological characteristics with TNBC (Choo & Nielsen 2010).

To the best of our knowledge, only scant literature has tried to explore the prevalence of BRCA 1/2 mutations among women with TNBC of different ethnic groups (Comen et al. 2011). Greenup et al. found that the prevalence of BRCA1 mutations was significantly higher among Ashkenazi Jewish women diagnosed with TNBC, as compared to African-American patients (Greenup et al. 2013). In Israel, a country with diverse and unique ethnic groups, there has been no attempt to characterize TNBC patients. The aim of the current study was to illustrate the clinical, molecular and prognostic characteristics of women with TNBC in Israel.

## Patients and methods

A retrospective review of the medical records of TNBC patients referred to a tertiary medical center (Rambam Health Care Campus, Haifa, Israel) between 1/1/2000 and 31/12/2005 was performed. Clinical data, molecular characteristics, treatment approaches and clinical data at follow-up visits were retrieved. Patients were divided into two main groups according to ethnicity: Arabic (A) and Jewish (J). ER, PR and HER2 status was extracted from routine pathology reports, and BRCA1/2 mutation status was obtained from genetic counseling reports. Follow-up clinical data included information about recurrent disease categorized as local, distant, or combined sites. Patients who presented initially with metastasis were not included in the recurrence parameters measurements.

### Statistical analysis

Statistical analysis was performed with SPSS package version 21. Descriptive statistics in terms of mean, median range, and percentiles were applied to all parameters. Quantitative parameters were summarized by mean ± standard deviation (SD) and examined using T-test or Mann–Whitney U test. Categorical parameters were summarized using frequency measures, and statistical analysis was performed using Fisher exact test. Survival analysis was performed with Log rank test. Cox regression model was used to determine the association between overall survival and several independent parameters. A p value of <0.05 was considered as statistically significant.

## Results

Overall, 122 consecutive TNBC patients were included in the study. The clinical, molecular, and histopathological characteristics of the TNBC patients are presented in Table 1. A majority of patients (74.6%) were younger than 60 years of age, and the median age was 54 years (range, 27–93 years). A total of 101 (82.8%) patients were Jewish (J) and 21 (17.2%) were Arabic (A).

**Table 1:** **Clinical, molecular and histopathological characteristics of triple-negative breast cancer patients**

**Characteristic**	**N**	**Percentage of total (%)**
**Age**		
>60 years	31	25.40
≤60 years	91	74.60
**No. of children**		
0	24	19.8%
1-3	66	54.5%
4+	31	25.6%
Missing	1	
**Ethnicity**		
Jewish	101	82.80
Arabic	21	17.20
**1st degree family history**		
Positive for breast cancer	30	24.60
Negative for breast cancer	92	75.40
**BRCA 1–2 mutation**		
Positive	22	73.33
Negative	8	26.67
**Histopathological type**		
Invasive ducal carcinoma	115	94.30
Medullary carcinoma	3	2.45
Infiltration lobular carcinoma	4	3.28
**Involved lymph nodes** ^**a**^		
Absent	78	66
1-3 nodes	27	23
≥4 nodes	13	11
**Tumor size**		
≤2 cm	66	54.00
>2.1 cm	56	46
**Surgical intervention** ^**b**^		
Lumpectomy	90	76.30
Mastectomy	28	23.7
**Adjuvant chemotherapy** ^**c**^		
Yes	85	81
No	20	19

**Notes**

^a^Exact number of involved lymph nodes missing in four patients.

^b^Four patients who had metastatic disease at presentation were not included in surgical and chemotherapy treatment.

^c^Adjuvant treatment data was missing for two patients, 11 patients received neoadjuvant treatment.

Reproductive history was examined according to the number of children at diagnosis and patients were divided into three subgroups, with the majority (54.5%) of TNBC patients having 1–3 children (Table 1).

With regard to inheritance, 1st degree family history for breast cancer was reported in 30 (24.6%) patients. Genetic counseling was conducted for these 30 patients, 22 of whom (73.3%) were positive for BRCA1/2 mutations. These patients represented 18% (22/122) of all the women included in the study.

Histopathological studies demonstrated that infiltrating ductal carcinoma (IDC) was the prominent histopathological type (115/122, 94.3%). Tumor size showed variability, with a median tumor size of 2 cm (range, 0–9 cm). Resected lymph nodes were positive for breast tumor by pathological examination in 40 (34%) patients.

Lumpectomy was the most common approach, performed in 76.6% patients. However, mastectomy was performed in nearly one-quarter of the patients.

Adjuvant chemotherapeutic treatment was given to 85 (81%) patients, while 11 patients received neoadjuvant therapy and, accordingly, were excluded from the treatment measurements.

Tumor progression was designated as metastasis development, local recurrence, or a combination of both. At the time of data analysis, 21/118 (17.8%) patients, who initially presented with early disease, had developed metastasis. Four patients who had metastatic disease at presentation were excluded. With regard to the location of the metastasis, the major metastatic sites included bone in 7 (33.3%), liver in 5 (23.8%), lung in 7 (33.3%), and central nervous system in 3 (14.3%). Local recurrence was detected in four (3.4%) patients. The time elapsed between diagnosis and recurrence was wide, with a median of 2.5 years (range, 0.25-11 years).

Within a follow-up period of 0.14-14 years following diagnosis, death of any cause was observed in 35 (28.70%) patients (Table 2).

**Table 2:** **Clinical outcomes of triple-negative breast cancer patients**

**Characteristic**	**N**	**Percentage of total (%)**
**Recurrence**		
Yes	25	21.18
No	93	78.88
**Pattern of Recurrence** ^**a**^		
Local	4	3.39
Distant	21	17.80
**Distant Metastasis**		
Yes	21	17.80
No	97	82.20
**Site of metastasis**		
Liver	5	23.80
Lung	7	33.3
Central nervous system	3	14.30
Bones	7	33.30
**Contralateral breast cancer**		
Yes	10	8.20
No	112	91.80
**Death of any cause**		
Yes	35	28.70
No	87	71.30

**Note**

^a^ Four patients had metastatic disease at presentation. These patients were not included in the recurrence measurements.

### Association of clinical parameters with overall survival (OS)

**Figure 1:**
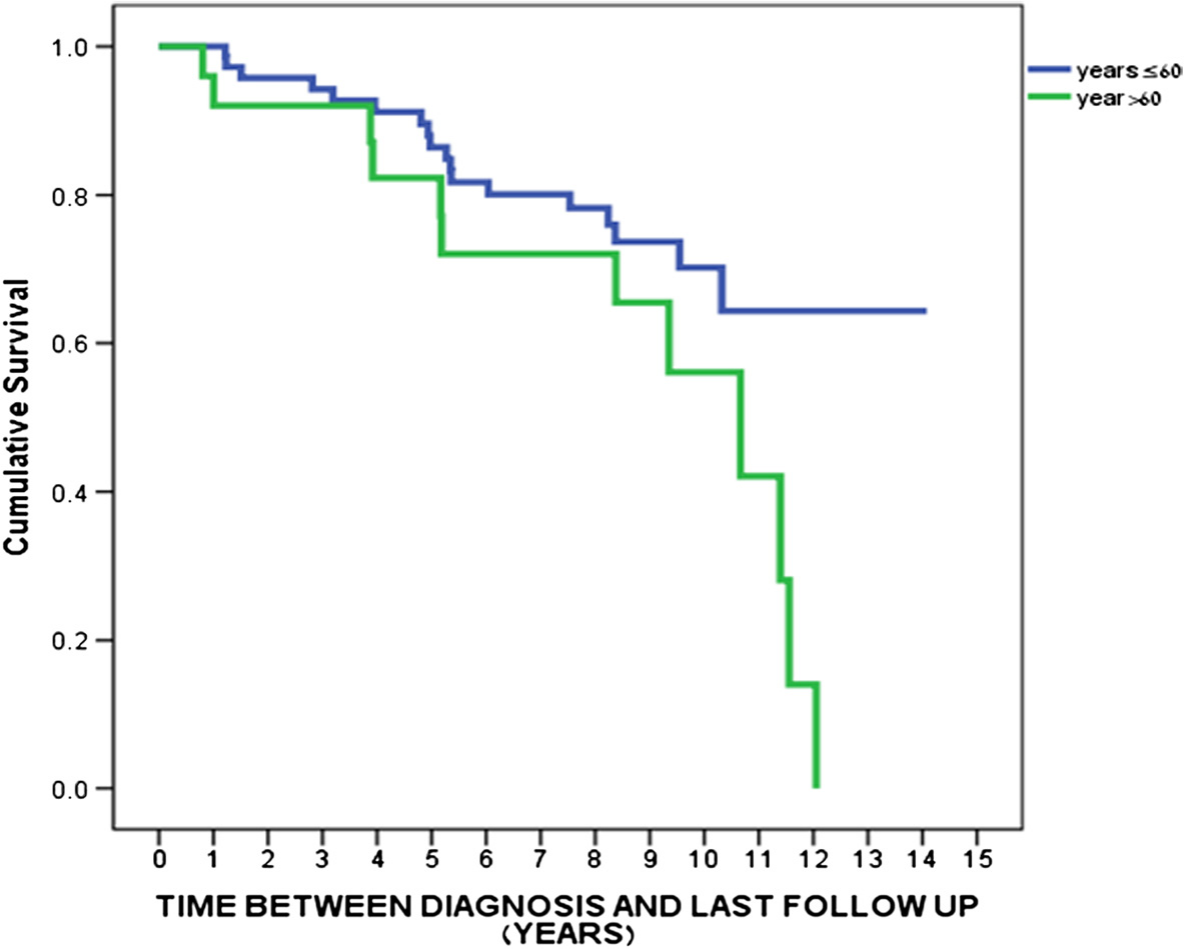
**The overall survival of TNBC patients stratified by age at diagnosis (p = 0.042).**

**Figure 2:**
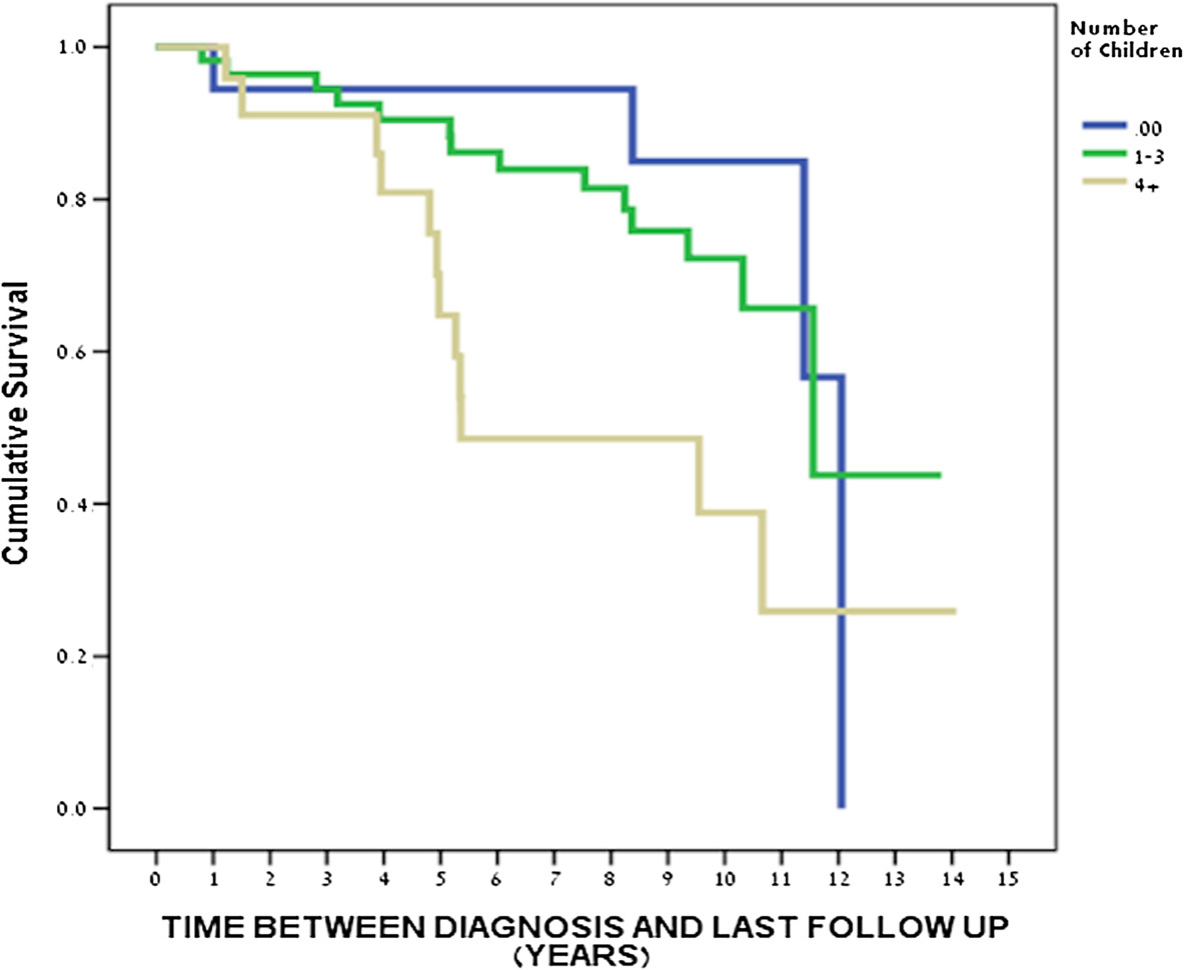
**The overall survival of TNBC patients stratified by number of children.** Between 0 to 4+: p = 0.033. Between 1–3 vs 4+: p = 0.012. Between 1–3 vs 0: p = 0.59.

We first explored the relationship between OS and different univariate analyses (Table 3). Interestingly, the cumulative survival was significantly higher for TNBC patients younger than 60 years, compared to those over 60 years (Figure 1). Notably, the overall survival for those with a reproductive history of ≥4 children was significantly lower than for TNBC patients without children. Similarly, the risk of mortality among TNBC patients with four or more children was higher compared to patients with 1–3 children (Figure 2).

**Table 3:** **Hazard ratio of overall survival and risk of death, according to different clinical and histopathological characteristics**

***Univariate Analysis***			
**Characteristic**	**Hazard ratio**	**95% confidence interval**	**p value**
**Age**			
>60 years vs ≤60 years	2.1	1.01-4.38	0.046
**No. of children**			
0 vs 4+	0.31	0.098-0.953	0.041
1-3 vs 4+	0.30	0.18-0.86	0.02
1+ vs 0	1.81	0.63-5.2	0.27
**Ethnicity**			
Arabic vs Jewish	1.33	0.40-4.42	0.64
**Family history**			
Positive vs negative for breast cancer	0.96	0.42-2.17	0.92
**Lymph nodes**			
Positive vs negative	2.45	1.07-5.60	0.033
**Tumor size**			
>2 cm vs ≤2 cm	1.46	0.68-3.10	0.33
**Surgery**			
Mastectomy vs lumpectomy	1.45	0.63-3.33	0.38
**Distant metastasis**			
Yes vs no	7.06	3.27-15.28	<0.001
***Multivariate Analysis***			
**Characteristic**	**95% confidence interval**		**p value**
**Age**			
>60 years vs ≤60 years	1.335-14.986		0.015
**No. of children**			
0 vs 1+	1.129-21.920		0.034

Additional data from multivariate analysis demonstrated that patients over 60 years of age had a higher mortality risk when compared to patients younger than 60 years of age (p = 0.015). Moreover a patient with one child or more had a higher risk of 4.9 folds for death when compared to women with no children (p = 0.034) (Table 3).

## Discussion

Several studies have illustrated the clinical and molecular characteristics of TNBC and showed that, unlike other breast cancer subtypes, TNBC is distinct because of lower disease-free survival, higher predisposition to form metastasis, and overall poor prognosis (Foulkes et al. 2010). We hereby provide the first study which aims to characterize TNBC patients in Israel according to molecular and clinical parameters. In the current study, we demonstrated that TNBC is more common among patients younger than 60 years. This finding is in accordance with previous literature. Dent et al. found that the mean age of TNBC women at diagnosis was 53 years (Dent et al. 2007) and Thike et al. reported a similar pattern with a median age of 52 years among TNBC at diagnosis (Thike et al. 2010), while other histopathological breast cancer subtypes are generally diagnosed at an older age (Boyle 2012).

We found that age younger than 60 years, nulliparity and absence of metastasis were associated with a statistically significant longer overall survival. It should be mentioned that only a few studies to date have examined age as an independent prognostic factor affecting clinical outcome among TNBC patients. Ovcaricek et al. reported a significantly higher risk of relapse for patients older than 65 years compared to those younger than 65 (Ovcaricek et al. 2011). Nevertheless, no significant differences were noticed in OS between the two groups.

There are very few data regarding the relationship between hormone receptor profile, including TNBC, and classical risk factors (Boyle 2012), with the assumption that differences in histopathological subtypes might explain the distinct or contradicted susceptibility of these subtypes to different risk factors.

Most interestingly, the interplay between parity status and TNBC incidence and clinical course was investigated. Phipps et al. showed a lower incidence of TNBC among nulliparous women, with the number of births positively correlated to the risk of TNBC (Phipps et al. 2011). These findings were corroborated by Nagatsuma et al. who reported a significant correlation between Japanese patients who had given birth most recently to higher rates of advanced stages of breast cancer, with an increased risk of negative estrogen and progesterone receptors and TNBC tumors when compared with nulliparous patients or those who had given birth less recently (Nagatsuma et al. 2013). Furthermore, significantly shorter overall survival rates were observed in patients with a more recent reproductive history when compared to patients with less recent parity (Nagatsuma et al. 2013). We showed that overall survival was significantly higher among nulliparous TNBC patients and correlated inversely with the number of births.

TNBC incidence varies among different ethnicities, suggesting that there are different genes that can predispose specific races to TNBC and distinct clinical behavior (Boyle 2012; Stead et al. 2009; Kurian et al. 2010; Amirikia et al. 2011; Huo et al. 2009). For instance, Stead et al. showed that TNBC was more prevalent among African-American women compared to White women (Stead et al. 2009). In addition, the lifetime risk of TNBC was highest among African-American women compared with Asian Hispanics and Whites in California (Kurian et al. 2010). In our study, however, ethnicity was not shown to affect overall survival. Although the distribution of the patients matched the percentages in the general population, the small number of Arabic patients (17.2%), where the majority was Jewish patients, might preclude obtaining significant different results according to ethnicity.

It is well-known that the majority of breast cancers that present in patients with positive BRCA1 mutations are TNBC (Atchley et al. 2008; Musolino et al. 2007). Moreover, recent studies showed that BRCA1-deficient tumors possess similar biological characteristics to TNBC (Choo & Nielsen 2010). Despite this significant clinical and biological association, the clinical ramifications and prognostic value of BRCA mutations in TNBC subgroups are not established yet (Lee et al. 2011). Lee et al. tried to illustrate the clinical outcomes according to BRCA status among TNBC patients. This study showed similar survival rates for carriers and non-carriers of BRCA mutations (Lee et al. 2011). In addition, a large study conducted by the Israel National Cancer Registration Center reported no significant differences among BRCA and non-BRCA patients of breast cancer (Rennert et al. 2007).

The prevalence of BRCA mutations among TNBC patients reported in the literature is 10-30% (Evans et al. 2011; Gonzalez-Angulo et al. 2011), while generally almost 10% patients diagnosed with breast cancer have a BRCA mutation (Frank et al. 2002). In this study, BRCA mutations were present in 22 patients (18% of all patients). Furthermore, BRCA mutation prevalence comprised a 53.3% (16/30) of patients with a 1st degree family history of breast and/or ovarian cancer. These current results coincide with earlier results on early onset breast cancer study in Israeli populations, showing BRCA mutations that reached 31% in entire group, but 57% in patients with a family history of breast and/or ovarian cancer (Gershoni-Baruch et al. 2000). However, to the best of our knowledge, our study is the first to report the prevalence of BRCA mutations among Israeli TNBC patients.

In Israel, genetic counseling is recommended for early-onset premenopausal Ashkenazi Jewish breast cancer patients and a familial history of breast or ovarian malignancy, regardless of histopathological subtype.

Kwon et al. reported significant results that supported routine testing of TNBC patients younger than 50 years as a cost-effective strategy, since this strategy provided an additional life expectancy for TNBC patients (Kwon et al. 2010). However, no worldwide validated policy exists yet.

Our study has several limitations. Firstly, the small cohort studies precluded the power to detect significant differences regarding the effect of race on the clinical behavior of TNBC patients. Secondly, only a small number of patients underwent genetic counseling and much smaller numbers were examined for BRCA1/2, thus precluding the ability to derive a conclusion about the clinical differences between BRCA and non-BRCA patients.

## Conclusions

We demonstrated that TNBC is more common among young patients below 60 years of age. The overall survival was significantly higher among nulliparous TNBC patients and correlated inversely with the number of births. Further prospective studies should reaffirm our findings and explore the effect of genetic profiling and ethnicity on the clinical course of TNBC patients.
